# Bilateral elbow joint osteonecrosis reconstructed by custom distal humerus hemiarthroplasty and megaprosthesis with tendon and nerve transfers – A case report

**DOI:** 10.1016/j.jpra.2026.01.039

**Published:** 2026-01-31

**Authors:** Yannick Albert J. Hoftiezer, Joseph O. Werenski, Carolina Posada Alvarez, Sang-Gil Lee, Santiago A. Lozano-Calderón

**Affiliations:** a Hand and Arm Service, Department of Orthopaedic Surgery, Massachusetts General Hospital, Yawkey Center Suite 2C, 55 Fruit Street, Boston, MA 02114, USA; b Department of Plastic, Reconstructive and Hand Surgery, Radboud University Medical Center, Geert Grooteplein Zuid 10, 6525 GA, Nijmegen, The Netherlands; c Orthopaedic Oncology Service, Department of Orthopaedic Surgery, Massachusetts General Hospital, Yawkey Center Suite 3B, 55 Fruit Street, Boston, MA 02114, USA; d Department of Orthopaedics, University Medical Center Groningen/University of Groningen, Hanzeplein 1, 9713 GZ, Groningen, The Netherlands; e Harvard Medical School, 25 Shattuck Street, Boston, MA 02115, USA

**Keywords:** Custom arthroplasty, Orthoplastic surgery, Osteonecrosis, Elbow, Tendon transfer, Nerve transfer

## Abstract

We present the case of a patient with bilateral distal humerus osteonecrosis following chemoradiation for lymphoma. Treatment consisted of a right total elbow arthroplasty with a distal humerus megaprosthesis and a left custom-made distal humerus hemiarthroplasty, replacing exclusively the humeral articular cartilage while preserving the native medial and lateral humeral columns. By using a custom implant of this design, we were able to avoid the weight-bearing and lifting restrictions to which patients have to adhere following a typical total elbow arthroplasty. Thus, we were able to preserve weight-bearing capacity in the dominant arm of the patient, which she required to act as a caregiver to a disabled family member. The case was complicated by a concurrent high radial nerve palsy which was treated by nerve decompression and transfers of tendons and nerves. Ultimately, bilateral upper extremity function was good with negligible pain at 2 years of follow-up. In conclusion, this case demonstrates the value of a combined orthoplastic approach and custom implants in successfully reconstructing complex upper extremity defects such as bilateral elbow joint osteonecrosis.

## Introduction

Osteonecrosis may arise from various etiologies such as steroid use, radiation, and alcoholism.^1^ Reduced bone quality secondary to osteonecrosis may result in fractures or osteoarthritis. In this case report, we describe our orthoplastic approach in a patient who developed bilateral distal humerus articular osteonecrosis which was complicated by a high radial nerve palsy.

## Case report

A 49-year-old patient presented to our service due to severe pain and decreased range of motion (ROM) in both elbows. Her medical history was remarkable for multifocal high-grade B-cell lymphoma including bilateral distal humerus, treated with chemoradiotherapy and steroids, as well as open reduction and internal fixation (ORIF) of a pathologic fracture of the right mid-humerus by double plating at a different institution.

**Figure 1:**
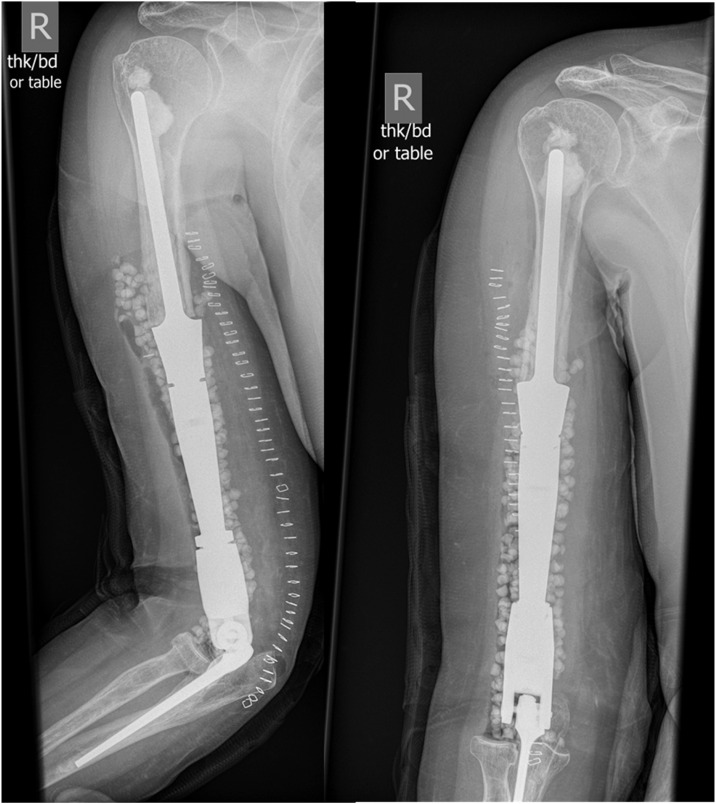
Perioperative imaging of right distal humerus replacement. Direct postoperative imaging studies of the right upper extremity following resection of distal humerus osteonecrosis and insertion of distal humerus endoprosthesis with total elbow arthroplasty. The implants are cemented-stemmed, with the humeral components consisting of a modular system with a large intercalary segment. Antibiotic-laden calcium sulfate beads are shown. There are no signs of complications, with the remaining bone being of sufficient quality without gross lucency or signs of osteonecrosis.

Radiographs of the right elbow demonstrated osteonecrosis of the capitulum with severely irradiated bone surrounding it. Therefore, resection of the distal humerus and reconstruction by total elbow arthroplasty with a distal humerus megaprosthesis was performed (Figure 1), with a hinged, semiconstrained and stemmed implant. (step-by-step methodology described in Supplemental Material 1).

The postoperative course was complicated by a severe right-sided radial neuropathy proximal to the triceps motor branch, partially secondary to radiation.

**Figure 2:**
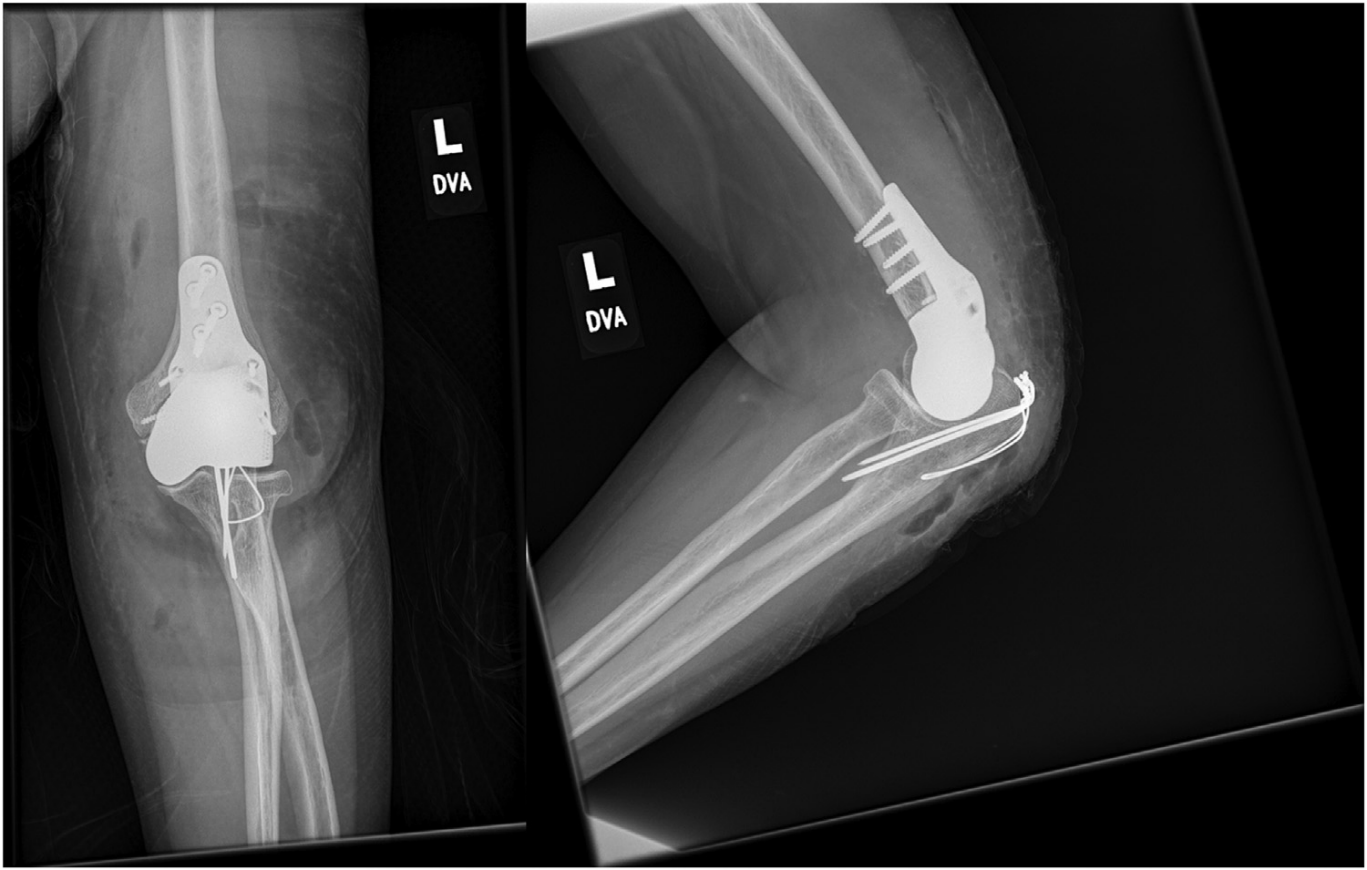
Postoperative imaging of left custom distal humerus hemiarthroplasty. Depicted here is the left elbow with the custom humeral implant a few weeks after placement. Note the relatively limited amount of resected bone as compared to the right-sided elbow allowing this specific design which preserves the medial and lateral columns of the distal radius and does not require a stemmed design. Tension band wiring of the olecranon is shown. There are no signs of complications, with the remaining bone being of sufficient quality without gross lucency or signs of osteonecrosis.

**Figure 3:**
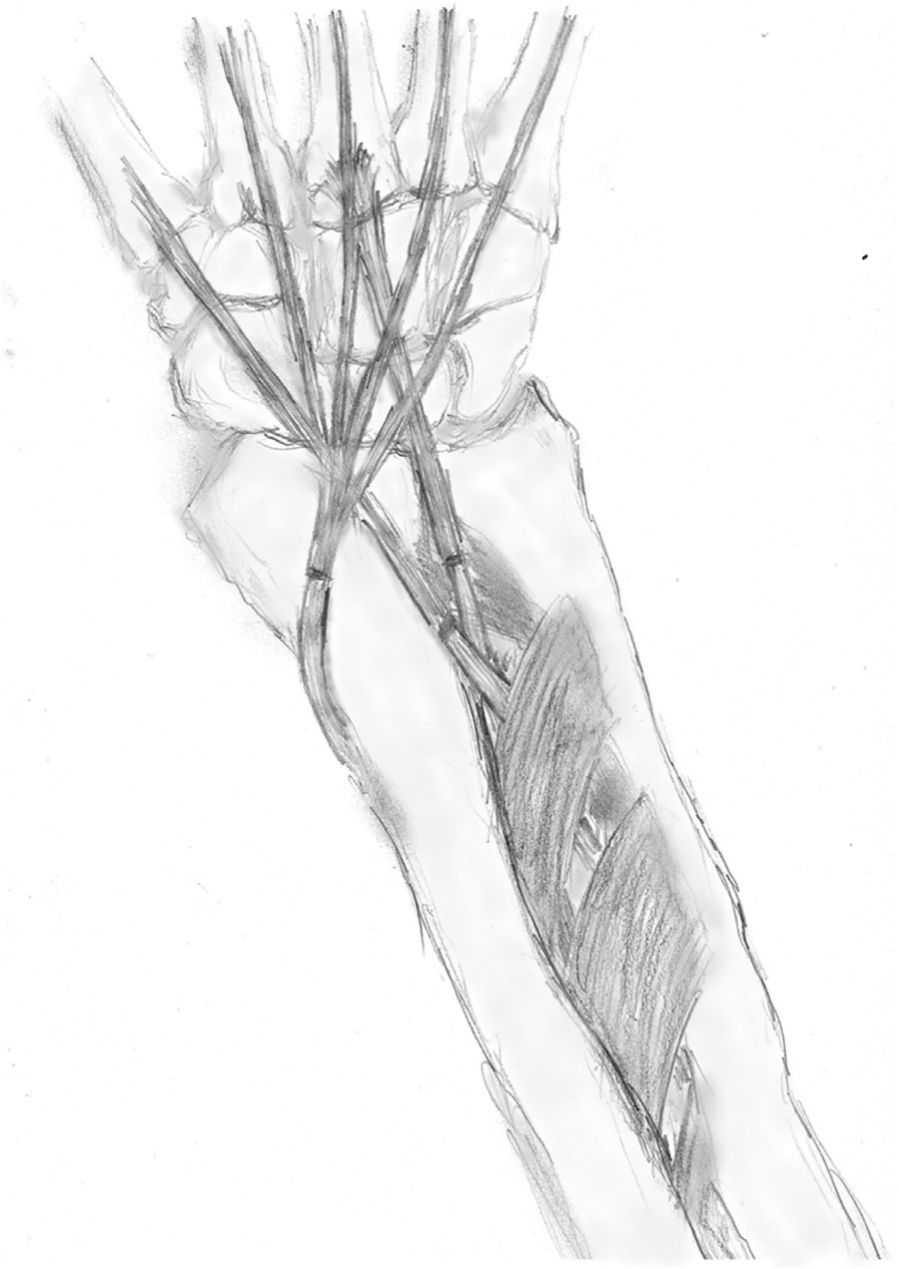
Artistic impression of right forearm tendon transfers. Depicted here are the forearm tendon transfers to mitigate the proximal radial nerve palsy. The tendons of the FDS of both the 3rd and 4th digit have been passed through the interosseous membrane and transferred to the ECRB and EPL, respectively. The FCR is coursed radially around the distal radius and transferred to the EDC tendons. All tendons were connected by means of a Pulvertaft weave which has not been drawn.

Radiographs of the left elbow demonstrated osteonecrosis of the articular area. Resection and replacement of the left distal humerus with a custom hemiarthroplasty were performed (Figure 2), with a stemless implant which was designed to only replace the affected articular surface of the distal humerus while preserving the native medial and lateral columns of the humerus (Supplemental Material 2). During the same procedure, transfers of tendons and nerve releases were performed to mitigate the effects of the right-sided radial nerve palsy (Figure 3). The flexor digitorum superficialis (FDS) of the 3rd digit was transferred to the extensor carpi radialis brevis (ECRB) to aid in wrist extension, while the FDS of the 4th digit was transferred to the extensor pollicis longus (EPL) to restore thumb extension. Furthermore, extension of the 2nd through 5th digits was restored by means of transferring the flexor carpi radialis (FCR) to the extensor digitorum communis (EDC). In order to restore elbow extension, the triceps was re-innervated by a pedicled nerve transfer of the flexor carpi ulnaris (FCU) motor branch from ulnar nerve motor branch to the triceps motor branch (Supplemental Material 3).

Tenolysis and tendon shortening subsequently further improved extension range and strength. At final follow-up, the patient was able to perform all activities of daily living, including acting as a caregiver to a disabled family member. The severe preoperative pain had decreased greatly. A complete overview of (patient-reported) outcome (measures) is presented in Table 1. In conclusion, the patient had good to excellent upper extremity function at 2 years of clinical follow-up.

**Table 1:** Clinical outcomes at latest follow-up.

	Left arm (dominant)	Right arm (non-dominant)
DASH	36.7	49.1
TESS UE	86.2	80.0
PROMIS upper extremity function	43.9	35.6
PROMIS physical function	46.6	44.4
Elbow extension	10 degrees deficit	45 degrees deficit
Elbow flexion	135	120
Wrist extension	complete	improving active extension sufficient for ADL
Wrist flexion	complete	complete
Digits extension	complete	improving active extension sufficient for ADL
Digits flexion	complete	complete
Weightbearing limit	none	5 pounds
	Overall	
PROMIS Pain Interference	47.9	

TESS UE, Toronto Extremity Salvage Score for the Upper Extremity; PROMIS, Patient-Reported Outcomes Measurements Information System; ADL, Activities of Daily Living; DASH, Disabilities of the Arm, Shoulder and Hand.

## Discussion

For the patient in this case report with complex bilateral upper extremity problems a multispecialty orthoplastic approach was selected to address both the osseous as well as motor nerve defects. The non-dominant extremity was reconstructed with a conventional cemented-stem, hinged and semi-constrained distal humerus replacement while the dominant extremity was treated with a custom made cementless distal humerus hemiarthroplasty implant while preserving the olecranon and both the medial and lateral columns of the native humeral bone. Nerve and tendon transfers were performed to restore motor function following proximal radial nerve palsy. Both arthrodesis and amputation were avoided, allowing the patient to preserve both upper extremities in a functional way.

Custom distal humeral replacements have been used for failed total elbow arthroplasty (TEA) and complex fracture patterns.^2^ In the left upper extremity of this patient, a conventional TEA or distal humeral replacement would have unnecessarily sacrificed a significant amount of bone, leaving the patient with the traditional deficits and restrictions in terms of weight-bearing in the dominant arm. Similar to TEAs, custom distal humeral replacements are available in linked (semi-constrained) and unlinked (unconstrained) designs. Depending on which structures require replacement, the design may incorporate segments to replace the distal humerus, humeral diaphysis, proximal ulna, radial head, and other segments of the elbow joint surface (e.g., the capitulum). In this case, the custom-made implant aimed to restore the humeral articular surface and preserve the olecranon and the medial and lateral columns of the humerus without weightbearing limits.

Due to the extensive osteonecrosis in the right elbow including the distal diaphysis and capitulum, the conventional distal humerus implant mostly resembling a hinged TEA with an elongated proximal intercalary component (to replace the humerus as far proximal as the mid-diaphysis) and intramedullary stem was elected. One major limitation of TEA or distal humerus replacement is the risk of aseptic loosening, which surgeons mitigate by imposing weight-bearing limits typically of 5 kg. However, due to the extensively diseased bone, a design of a similar type to the left upper extremity was not feasible.

Neurovascular integrity is crucial for achieving functional limb outcomes, alongside osseus stability and articular surface congruity. The complete and proximal radial nerve palsy in the right arm of the patient severely limited the function of her reconstructed limb. When nerve regeneration is not expected, options for functional restoration typically involve tendon, nerve, or muscle transfers.^3^, ^4^, ^5^, ^6^, ^7^ To address the lack of wrist and digital extension, tendon transfers from volar (flexor) compartment muscles to the dorsal (extensor) compartment muscles were performed.^4^ Options to replace triceps muscle for elbow extension are limited and thus a pedicled nerve transfer of the flexor carpi ulnaris motor branch to the triceps motor branch was performed.^8^

Custom or patient-specific implants have recently become more common, especially in cases with complex oncologic or traumatic reconstructions. Some of the purported advantages over regular or modular implant systems are the ability to achieve both a perfect fit with retained native bone (in this case of the proximal humerus) and improved joint congruency, with the improved interface with the native bone decreasing the chances of loosening.^9^ Their method of design typically involves obtaining a CT scan of the contralateral bone or joint and mirroring this, resulting in a 3D design that allows an accurate replacement of the affected bone.^10^ For the custom implant, the resection of affected bone was aided by a matched cutting guide, while the implant had a trabecular configuration at the interface between implant and resection surface.

For both extremities amputation or arthrodesis could be considered but these would result in loss of function of the hand and elbow or only the elbow, respectively. Arthrodesis would carry a significant risk of non-union or fracture due to the remaining osteonecrotic bone. After thorough discussion with the patient, functional reconstruction of both extremities was elected.

This report describes a treatment strategy for the complex and uncommon problem of bilateral distal humerus osteonecrosis complicated by radial nerve palsy. The orthoplastic approach resulted in good to excellent upper extremity function, resulting in only slight limitations in daily life and restored her ability to act as a caregiver for a disabled family member.

## Funding

None.

## Ethical approval

Not required, although the patient presented in this case report explicitly agreed to publication of this material.

## Declaration of competing interest

None declared.

## Appendix Supplementary materials

Image, application 1

Image, application 2

Image, application 3
