# Supplementary material for: Bilateral elbow joint osteonecrosis reconstructed by custom distal humerus hemiarthroplasty and megaprosthesis with tendon and nerve transfers – A case report
Source: JPRAS Open. 2026 Jan 31;49:35–40. doi: 10.1016/j.jpra.2026.01.039 (PMC12938139; doi:10.1016/j.jpra.2026.01.039)
Supplement: Supplementary file 1 [file mmc1.docx]

**Supplemental Material 1 – Step-wise explanation of right distal humerus megaprosthesis placement**

The patient was induced under general anesthesia in the OR and received an additional nerve catheter for postoperative pain control as well as preoperative antibiotics. The patient was placed in the lateral decubitus position, sterilized and draped in a standard fashion. Both the existing incisions (volar and dorsal) from prior double plating of the distal humerus were opened in order to expose the current hardware. The dorsal incision was made over the distal humerus was followed by a lateral tricipital approach and mobilizing the triceps medially to expose the distal humerus. Both plates were removed uneventfully with attention towards protection of the radial nerve. The humerus was inspected and found to contain osteonecrosis as far proximally as the mid-diaphysis while the visible parts of the proximal ulna and radius were in good condition. Thus, the affected segment of the humerus was dissected free from soft tissues and then cut proximally and then removed. Medullary canals in the remaining segment of the humerus as well as the proximal ulna were prepared under fluoroscopy and cemented stems were inserted after which the conventional stemmed humerus endoprosthesis and total elbow arthroplasty were constructed in a standard fashion. Congruency and implant position were confirmed under fluoroscopy and function of the elbow was tested passively for range of motion and to exclude any impingement. The operative field was then irrigated after which antibiotic-laden calcium sulfate beads (vancomycin and tobramycin) were inserted. The wounds were closed in layers preserving the collateral ligaments and remaining joint capsule, no drains were used. Note that the triceps was only mobilized and not transected and thus did not require any repair. The arm underwent sterile dressing and was placed in a sling for comfort. Range of motion was allowed as tolerated but weightbearing was restricted at 5 pounds. The patient received postoperative antibiotics for 24 hours.
