# Supplementary material for: Bilateral elbow joint osteonecrosis reconstructed by custom distal humerus hemiarthroplasty and megaprosthesis with tendon and nerve transfers – A case report
Source: JPRAS Open. 2026 Jan 31;49:35–40. doi: 10.1016/j.jpra.2026.01.039 (PMC12938139; doi:10.1016/j.jpra.2026.01.039)
Supplement: Supplementary file 2 [file mmc2.docx]

**Supplemental Material 2 – Step-wise methodology of left distal humerus custom implant placement**

The patient was induced under general anesthesia in the OR and received an additional nerve catheter for postoperative pain control. The left arm was placed on an arm table, the patient was sterilized and draped in a standard fashion. A longitudinal incision over the medial aspect of the elbow was performed and following dissection of the subcutaneous tissues the ulnar nerve was mobilized along with the distal part of the medial intermuscular septum in the distal arm. Following mobilization of the ulnar nerve the medial aspect of the elbow joint was exposed using a medial paratricipital approach after which a chevron osteotomy of the olecranon was made using an oscillating saw. The proximal olecranon along with the insertion of the triceps were reflected proximally after dissecting the triceps from the anconeus muscle, thereby completing the exposure of the distal humerus. A custom cutting guide, which was designed alongside the custom implant, was temporarily secured with 3 K-wires and then used to resect the affected segments of the distal humerus as a single specimen after dissecting off any remaining soft tissues. The custom implant was inserted; excellent restoration of the articular surface and bone-implant contact were achieved. The implant was secured to the humeral shaft with 3.5mm screws and to the lateral and medial columns of the humerus with 2.7mm screws, all ranging 18-22mm in length. The deflected part of the proximal olecranon was relocated to its original position and fixated using tension band wiring with two 2.6mm K-wires along with a 18 gauge wire. Fluoroscopy in multiple directions was performed to confirm excellent reduction of the olecranon osteotomy, as well as implant position and joint congruency. The ulnar nerve was transposed anteriorly using a standard subfascial technique; the nerve was confirmed to be without tension, compression or kinking. Copious irrigation was performed. The wound was closed in layers without a drain, underwent sterile dressing, after which the arm was immobilized in a sling. Antibiotic prophylaxis by vancomycin was administrated for 24 hours. The left arm was to remain non-weightbearing although passive ROM and assisted active ROM without axial loading were allowed.
