# Supplementary material for: Bilateral elbow joint osteonecrosis reconstructed by custom distal humerus hemiarthroplasty and megaprosthesis with tendon and nerve transfers – A case report
Source: JPRAS Open. 2026 Jan 31;49:35–40. doi: 10.1016/j.jpra.2026.01.039 (PMC12938139; doi:10.1016/j.jpra.2026.01.039)
Supplement: Supplementary file 3 [file mmc3.docx]

**Supplemental Material 3 – Step-wise methodology of right tendon and nerve transfers**

This procedure was performed simultaneously with the left-sided custom distal humerus implant. The patient was induced under general anesthesia in the OR and received an additional nerve catheter for postoperative pain control. The right arm was placed on an arm table, the patient was sterilized and draped in a standard fashion. Longitudinal incisions over the volar (12cm) and dorsal (10cm) aspects of the forearm were made in order to expose FDS3-4 and ECRB/ECRL, EPL and EDC’s respectively. The FDS3 was transected with sufficient length to be rerouted through the interosseous membrane and reach the ECRB tendon. Using the Pulvertaft weave technique, the FDS3 was then sutured with 3-4 weaves into the tendinous parts of the ECRB. Non-resorbable braided 2-0 sutures were used. Appropriate tension was determined by ensuring that the tendon transfer passively held the wrist in slight extension. Similarly, the FDS4 was rerouted through the interosseous membrane and sutured (again using the Pulvertaft technique) to the EPL in order to bring the thumb in a position of slight passive extension. Note that the native musculotendinous junction of the EPL was transected to improve the line of pull of the FDS4-EPL transfer before suturing and determining the amount of tension needed. All EDC tendons to digits 2-5 were isolated on the dorsal wrist after which the FCR was dissected out from the radial volar wrist and rerouted subcutaneously around the radial aspect of the forearm. Then the FCR was sutured into the EDC2-5 complex by Pulvertaft weave slightly proximal to the dorsal wrist, tension was determined by aiming to restore the natural cascade of the digits.

After completion of the tendon transfers, attention was directed towards the nerve transfer to restore triceps function. First, an 8cm incision was made in the axillary region to expose and identify the individual divisions and cords. Upon identification of the ulnar and radial nerves using a standard neurostimulator, dissection of the ulnar nerve was continued on a fascicular level to identify a fascicle with significant FCU motor function. This fascicle was freed both proximally and distally from the remaining ulnar nerve fascicles to gain sufficient length for anastomosis with a motor branch to the triceps muscle, after which the FCU motor fascicle was transected distally. The posterior cord was identified and followed distally towards some of the triceps motor branches of the radial nerve. Upon identification of a significant triceps motor branch this was transected proximally and under microscope magnification the nerve transfer was completed in an end-to-end fashion using 9-0 nylon sutures covered by fibrin glue. All wounds were irrigated, closed in layers without the use of drains and underwent sterile dressing. The hand and digits were placed in an extension splint. The splint was to be worn continuously besides during guided range of motion exercises by a hand therapist. The elbow and shoulder were allowed to be used and moved as tolerated by the patient.
